# Extracellular signal-regulated kinase in the basolateral amygdala is required for reconsolidation of heroin-associated memory

**DOI:** 10.3389/fnmol.2022.1020098

**Published:** 2022-11-10

**Authors:** Haoyu Li, Ting Hu, Yanghui Zhang, Zijin Zhao, Qing Liu, Zihua Chen, Si Chen

**Affiliations:** ^1^Department of Neurosurgery, Xiangya Hospital, Central South University, Changsha, China; ^2^The Institute of Skull Base Surgery and Neurooncology at Hunan Province, Changsha, China; ^3^National Clinical Research Center for Geriatric Disorders, Xiangya Hospital, Central South University, Changsha, China; ^4^Center of Medical Genetics, Jiangmen Maternity and Child Health Care Hospital, Jiangmen, China; ^5^Department of General Surgery, Xiangya Hospital, Central South University, Changsha, China; ^6^Department of Ophthalmology, Xiangya Hospital, Central South University, Changsha, China; ^7^Hunan Key Laboratory of Ophthalmology, Changsha, China

**Keywords:** heroin, reconsolidation, ERK, BLA, relapse

## Abstract

Reconsolidation of heroin-associated memory is an independent memory process that occurs following retrieval, which is essential for the sustained capacity of an associative drug stimulus to precipitate heroin-seeking. Extracellular signal-regulated kinase (ERK) in the basolateral amygdala (BLA) mediates the reconsolidation of drug memory. In the present study, we utilized a rat model of drug craving and relapse to verify the hypothesis that the reconsolidation of heroin-associated memory requires ERK in an instrumental heroin-seeking behavior, focusing on the BLA brain region, which is crucial for synaptic plasticity and memory processes. We found that bilateral intra-BLA infusions of U0126 (1 μg/0.5 μl), an ERK inhibitor, immediately after retrieving heroin-associated memory significantly reduced cue-induced and drug-induced reinstatement and spontaneous recovery of heroin-seeking compared to the vehicle. Furthermore, this inhibitory effect was related to the characteristic of reconsolidation. Conversely, no effect was observed on the heroin-seeking behavior when the intra-BLA infusion of U0126 was administered 6 h after the heroin-associated memory retrieval or without memory retrieval. Together, these data suggest that disrupting the reconsolidation of heroin-associated memory *via* an ERK inhibitor may serve as a promising option for treating relapse in opiate addicts.

## Introduction

The associative memory formed by repeated drug use usurps standard reward-related memory, leading to substance-related and addictive disorders that result from a disturbance in learning, emotional, decision-making, and response systems (Gardner, 2011; Volkow et al., 2019). Such aberrations lead addicts to reemerge with compulsive drug-seeking behavior and even relapse (Hyman et al., 2006; Gardner, 2011). Exposure to environmental cues or contexts associated with drug elicits craving and relapse, which is the core primary clinical problem in drug addicts (Childress et al., 1999; Crombag et al., 2008; Chen et al., 2021b). To address the persistent propensity for relapse, an increasing number of studies suggest that disrupting drug-associated memory, which maintains conditioned reinforcing properties through various manipulations, is a promising strategy to prevent relapse (Milton and Everitt, 2010; Jian et al., 2014; Barak and Goltseker, 2021).

The memory reconsolidation supposes the hypothesis that the consolidation memory underwent retrieval *via* reexposure to the conditioned stimulus (CS) and becomes labile during a time window. It requires a *de novo* protein synthesis-dependent reconsolidation process to subsist (Nader et al., 2000; Kida et al., 2002; Tronel et al., 2005; Fukushima et al., 2014; Chen et al., 2021a; Zhang et al., 2021). Disrupting the reconsolidation of drug memory would limit relapse susceptibility. Studies in animal models and human addicts find that intervening in the reconsolidation of drug memory subsequently attenuates drug-seeking behaviors (Milton and Everitt, 2010; Sorg, 2012; Liang et al., 2017; Lin et al., 2021). Therefore, elucidating the molecular mechanism of reconsolidation is of great significance for developing a highly selective drug therapy to prevent relapse.

The mitogen-activated protein kinase (MAPK) ERK is essential for synaptic plasticity, learning, and memory, which is especially involved in the reconsolidation of some memory forms (Lu et al., 2006; Peng et al., 2010; Leal et al., 2014). ERK activation in neurons significantly integrates signaling for the reconsolidation of drug memory, including N-methyl D-aspartate receptors (NMDARs; Brown et al., 2008; Milton et al., 2008), the activation of β-adrenergic receptor, and the stimulation of protein kinase A (PKA; Sweatt, 2001; Fricks-Gleason and Marshall, 2008; Milton and Everitt, 2010; Sanchez et al., 2010). Furthermore, studies suggest that ERK is critical for the reconsolidation of auditory fear memory (Duvarci et al., 2006), object recognition memory (Kelly et al., 2003; Silingardi et al., 2011), and cocaine memory in both Pavlovian cocaine-related memory of the conditioned place preference (CPP) model and response-outcome associative cocaine-related memory of the self-administration (SA) model (Miller and Marshall, 2005; Valjent et al., 2006; Wells et al., 2013). Nevertheless, several gaps remain in our understanding of the effect of ERK on the memory reconsolidation. More specifically, the effect of ERK on the reconsolidation of heroin-associated memory has not been investigated in the response-outcome paradigm, since studies show that the reconsolidation of drug memory in Pavlovian and instrumental patterns involves distinct neuroanatomical mechanisms (Wells et al., 2016; Bender and Torregrossa, 2020).

In the present study, we used the classical heroin SA model of relapse to confirm whether ERK is required for the reconsolidation of heroin-associated memory in the brain regions of the BLA that have been demonstrated to critically regulate reconsolidation of drug memory (Li et al., 2010; Sanchez et al., 2010; Xie et al., 2022). We also tested the effect of ERK inhibition during reconsolidation on subsequent cue-induced and heroin-induced reinstatement and spontaneous recovery of heroin-seeking behavior.

## Methods

### Subjects

Male Sprague-Dawley rats (260–280 g on arrival) were placed in a 23 ± 2°C temperature and 50% humid environment with free access to food and water under a 12-h light/dark cycle. The rats were handled for 3 min/day for 5 days before surgery. All experimental procedures were performed under the Guidelines of the Xiangya Hospital Ethics Committee, Xiangya Hospital (Changsha, China).

### Surgery

**Figure 1:**
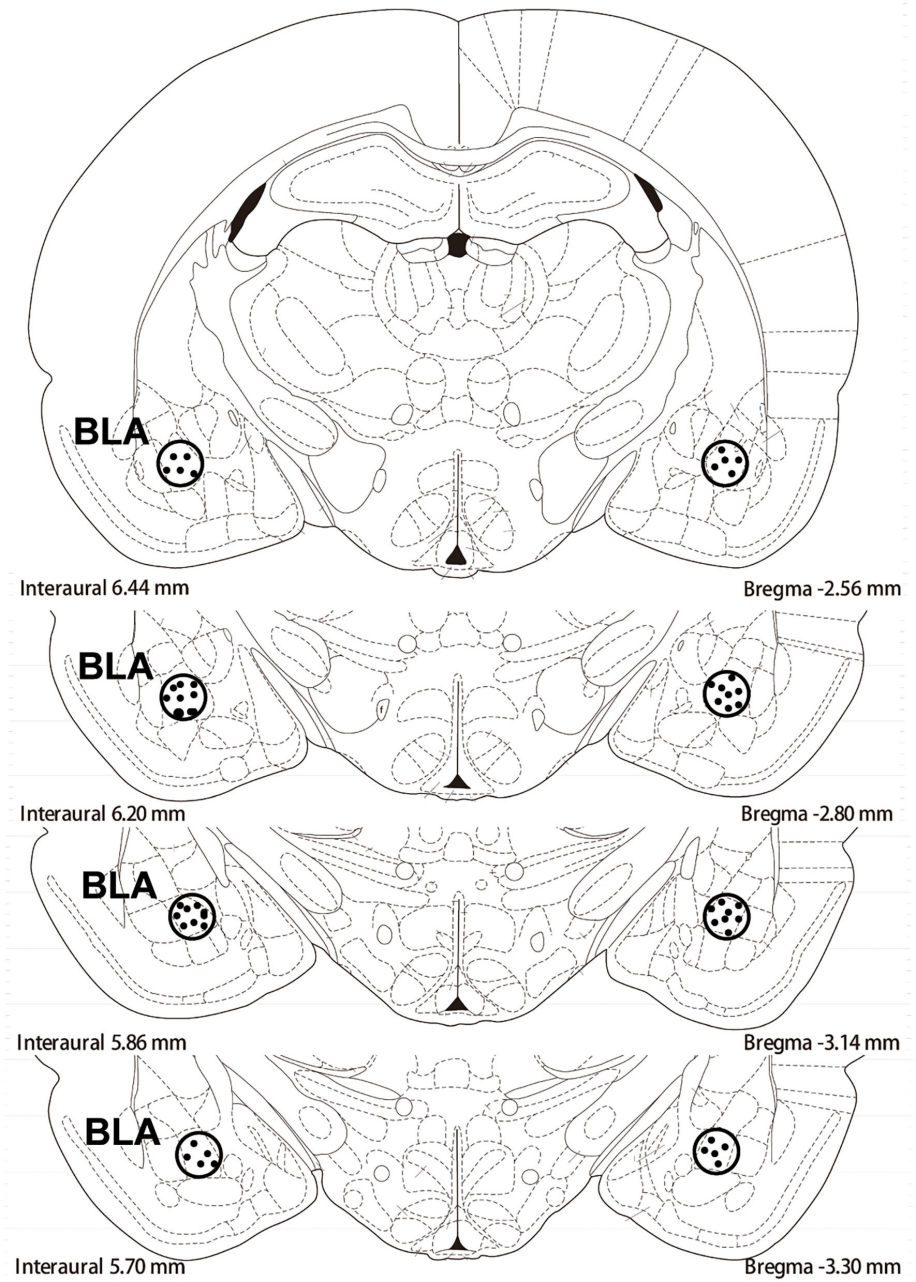
The schematic depiction of the region basolateral amygdala (BLA) of cannula placements: −2.8 mm from bregma.

After 60 mg/kg of sodium pentobarbital anesthesia was administered intraperitoneally (ip) to rats, the rats received an implantation with intravenous (iv) jugular catheters and bilateral guide cannulae (23 gauge; Plastics One, Roanoke, VA, USA) surgically (Xue et al., 2012; Xie et al., 2022). The implantation region of the guide cannulae was 1 mm above the BLA (anterior/posterior:−2.8 mm, medial/lateral: ±5.0 mm from bregma, and dorsal/ventral: −8.5 mm from the surface of the skull; Wu et al., 2011; Xie et al., 2022). Then, the animals received 5–7 days of recovery. The regions of the representative cannula placements in the basolateral amygdala as shown in the rostral faces of each coronal section (see Figure 1).

### Behavioral procedures

#### Heroin SA training

Heroin SA training procedures were established in previous studies (Xue et al., 2012; Luo et al., 2015). Two nosepoke operandi (active/inactive) were placed 9 cm above the floor of the chambers (AniLab Software and Instruments Co., Ltd., Ningbo, China). As a result of nosepoke into the active operandum, rats received heroin infusions paired with tone/light cues for 5 s. As a result of nosepoke into the inactive operandum, there was no consequence.

The heroin SA training session was carried out every 10 days, and the rats received three 1-h training sessions separated by 5 min. The training used a 1:1 fixed-ratio reinforcement schedule at the start of each dark cycle. Every infusion was followed by a 40 s period that had no consequence of nosepoke. The house light was turned on at each session. A maximum of 20 heroin infusions per hour were permitted (Xue et al., 2012; Luo et al., 2015).

#### Nosepoke extinction

Rats then underwent extinction training in the original environment for 10 days with no illumination or stimulation after SA in all four experiments. In this situation, nosepoke (active/inactive) resulted in no heroin infusion or tone/light cue consequences.

#### Heroin reward memory retrieval

A 15-min reactivation experiment was conducted 24 h after the last extinction session under conditions that were similar to those for SA training, except no heroin infusions occurred following active nosepoke (experiments 1, 2, and 4).

#### U0126 treatment

Immediately after retrieval, U0126 was infused bilaterally (1.0 μg/0.5 μl/side for 2 min; Calbiochem) into the BLA of rats (experiments 1 and 2; Wells et al., 2013). The 10 μl Hamilton syringes were linked to the 28-gauge infusion cannulae (Plastics One). An equal volume of 5% dimethyl sulfoxide (DMSO) was infused into the control rats. In experiment 3, infusions of U0126 or vehicle were administered to rats without reactivating light/tone stimuli. In experiment 4, we infused rats with U0126 or vehicle 6 h after retrieval.

#### Cue extinction

The cue extinction conditions were the same as those during heroin SA sessions, except for the absence of heroin infusions after the tone/light cue. For experiments 1, 3, and 4, rats were subjected to a 3-h daily cue extinction.

#### Cue-induced reinstatement test (experiments 1–4)

Twenty-four hours after U0126 or vehicle infusion into the BLA, rats were subjected to this test. The conditions were the same as those for the SA, except that the active nosepoke had contingent tone-light cues but without heroin infusions. The number of active and inactive nosepokes was recorded for one hour.

#### Heroin-induced reinstatement test (experiments 1, 3, and 4)

After the 5-min heroin infusion [0.25 mg/kg, subcutaneously (sc)], rats were subjected to heroin-induced reinstatement. Conditions were the same as those for the SA, except that active nosepoke was paired with tone/light cues but not heroin. The number of active and inactive nosepokes was recorded for 1 h.

#### Spontaneous recovery test (experiment 2)

After 28 days of withdrawal, the number of nosepokes (active and inactive) was recorded for 1 h in the same condition as reactivation.

### Specific experiments

#### Experiment 1

The role of immediate post-CS ERK inhibition in the BLA in the subsequent cue-induced and heroin-induced reinstatement of heroin-seeking behavior.

**Figure 2:**
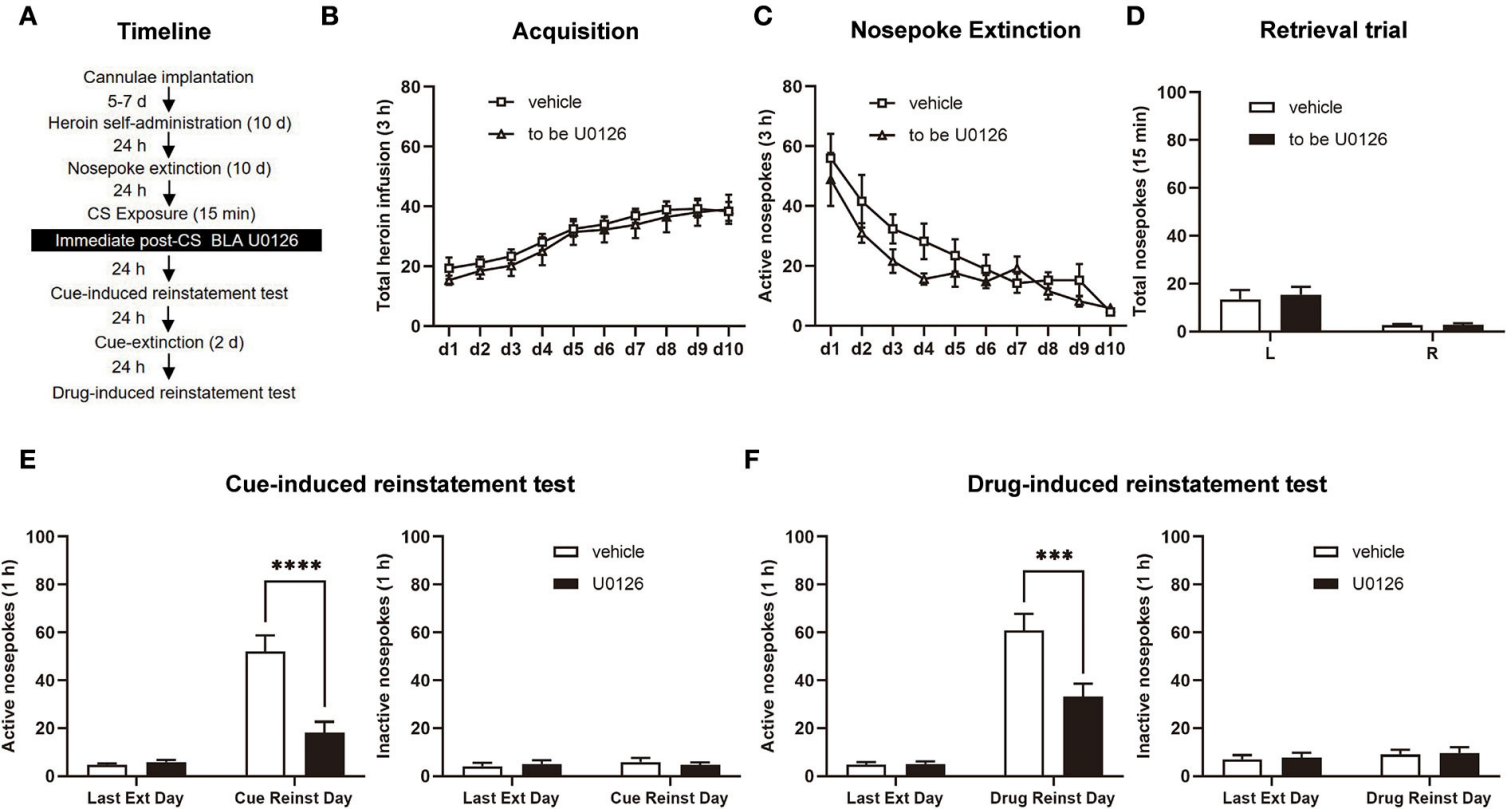
Immediate post-CS (conditioned stimulus) U0126 intra-BLA (basolateral amygdala) treatment reduces subsequent cue-induced and heroin-induced reinstatement of heroin seeking. **(A)** Schematic depiction of the experimental procedure. **(B)** Total number of heroin infusions during the acquisition of heroin self-administration. **(C)** Active nosepoke responses during extinction training. **(D)** Total nosepoke responses during a retrieval trial. **(E)** Active **(left)** and inactive **(right)** nosepokes during the last extinction session and cue-induced reinstatement test. **(F)** Active **(left)** and inactive **(right)** nosepokes during the last extinction session and heroin-induced reinstatement test. *n* = 8 rats per group. Data are means ± SEM, ***p < 0.001, ****p < 0.0001, compared with the vehicle group. CS, conditioned stimulus; Ext, extinction; Reinst, reinstatement.

Following the 10-day heroin SA training sessions, the rats received 9 days of nosepoke extinction in the original environment. Twenty-four hours after the last nosepoke extinction, heroin-associated cues were presented for 15 min to reactivate the drug-associated memory. Immediately after retrieval, one group of rats was infused with U0126 intra-BLA bilaterally at 1.0 μg/0.5 μl/side, termed the U0126 group, and another group of rats was infused with vehicle intra-BLA bilaterally at 0.5 μl/side, termed the vehicle group. Twenty-four hours after infusion, a cue-induced reinstatement test was carried out to investigate heroin-seeking behavior in rats. Then, we tested priming-induced reinstatement after 2 days of cue extinction (see Figure 2A).

#### Experiment 2

The role of immediate post-CS ERK inhibition in the BLA in subsequent cue-induced heroin seeking and spontaneous recovery of heroin-seeking behavior after long-term (28 days) withdrawal.

**Figure 3:**
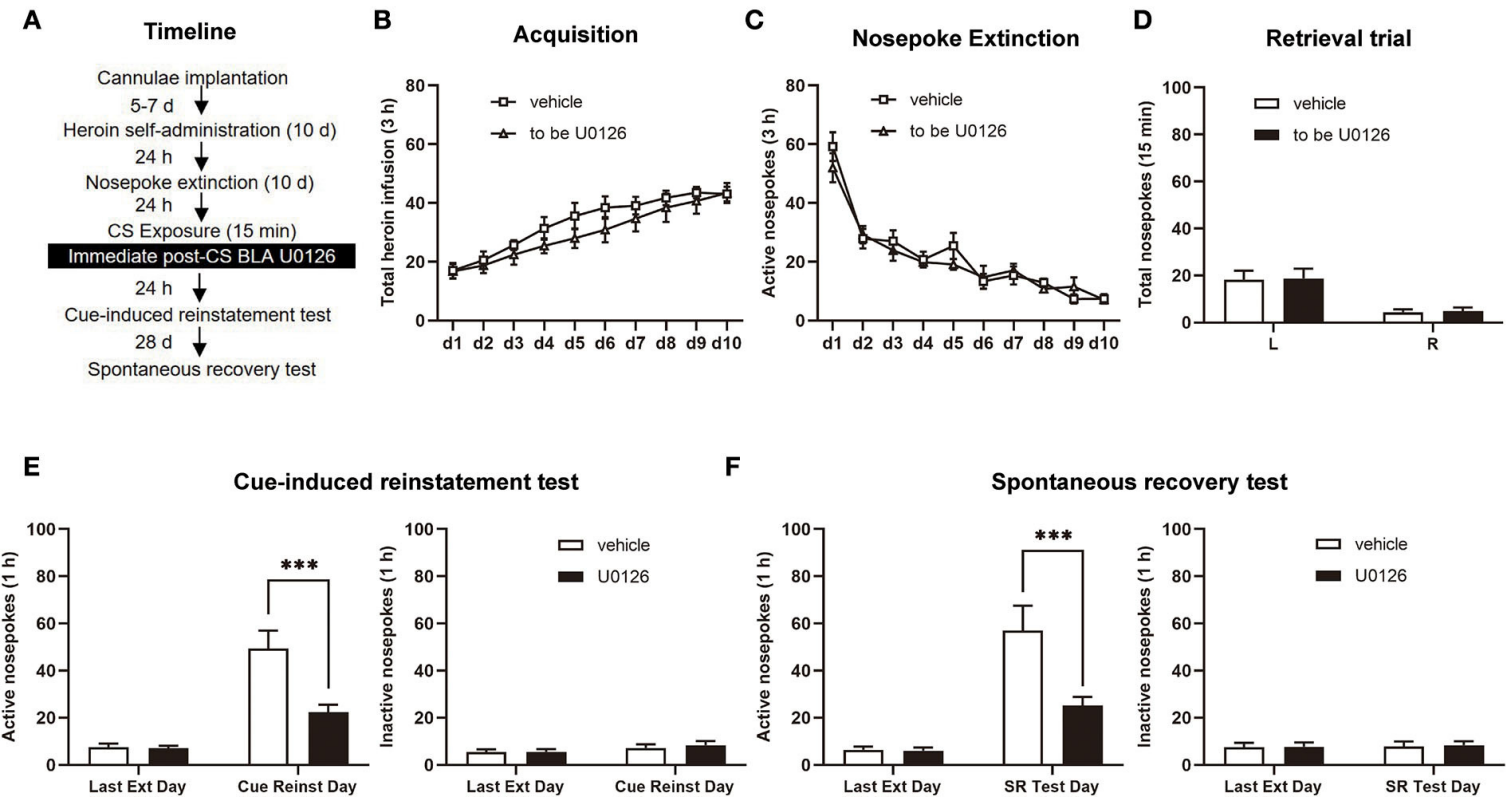
Immediate post-CS (conditioned stimulus) U0126 intra-BLA (basolateral amygdala) treatment reduces subsequent cue-induced heroin seeking and the spontaneous recovery of heroin-seeking behavior. **(A)** Schematic depiction of the experimental procedure. **(B)** Total number of heroin infusions during the acquisition of heroin self-administration. **(C)** Active nosepoke responses during extinction training. **(D)** Total nosepoke responses during a retrieval trial. **(E)** Active **(left)** and inactive **(right)** nosepokes during the last extinction session and cue-induced reinstatement test. **(F)** Active **(left)** and inactive **(right)** nosepokes during the last extinction session and spontaneous recovery test. *n* = 9 rats per group. Data are means ± SEM, ***p < 0.001, compared with the vehicle group. CS, conditioned stimulus; Ext, extinction; Reinst, reinstatement; SR, spontaneous recovery.

After heroin SA and nosepoke extinction sessions (same as Experiment 1), rats were allowed to be infused with U0126 (1 μg/0.5 μl/side), and the control group of rats was infused with an equal volume of vehicle following retrieval. Twenty-four hours later, a cue-induced reinstatement test was performed. After 28 days of withdrawal, we tested spontaneous recovery in rats (see Figure 3A).

#### Experiment 3

The role of ERK inhibition in the BLA without retrieval in subsequent cue-induced and heroin-induced reinstatement of heroin-seeking behavior.

**Figure 4:**
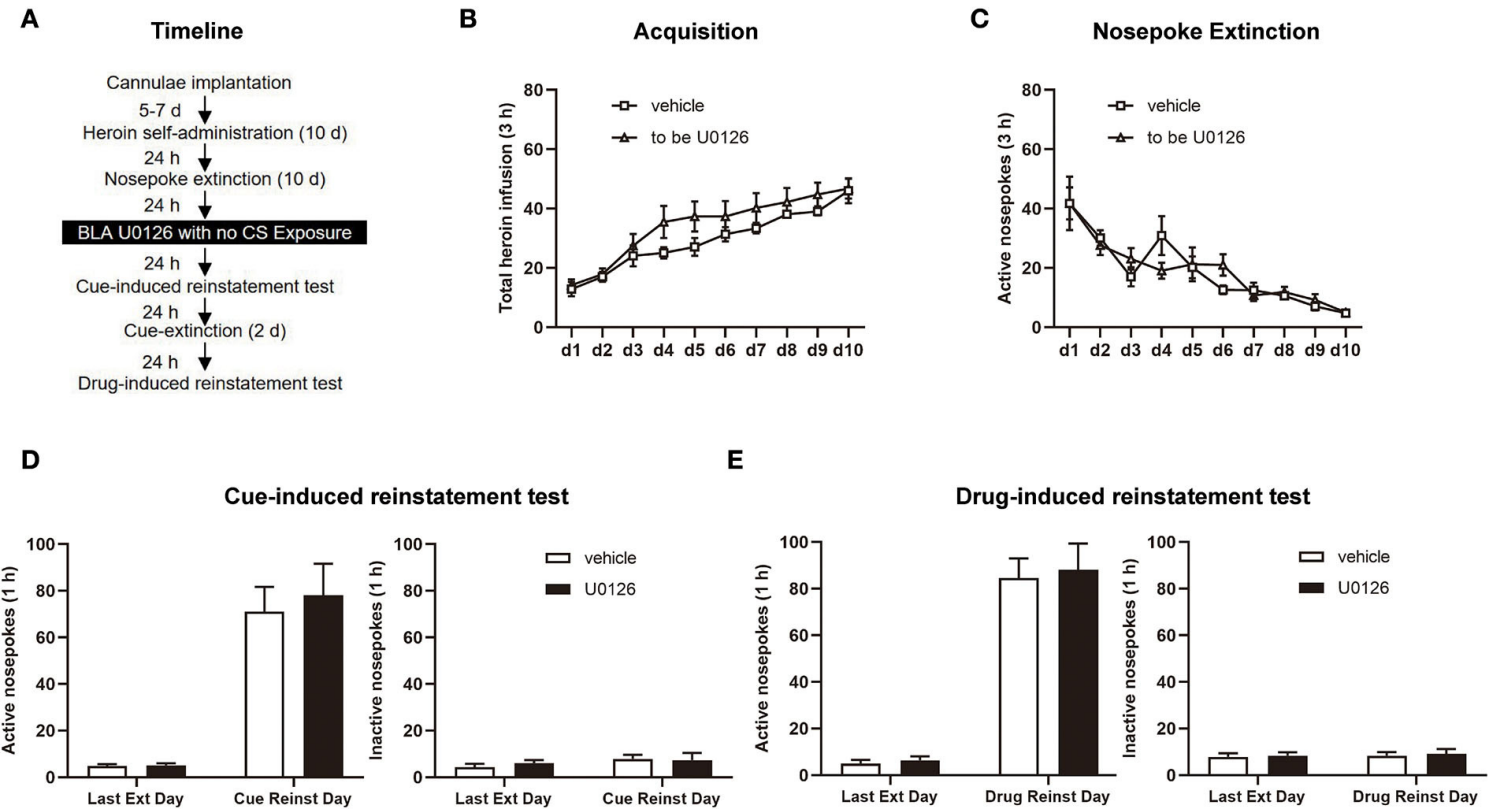
U0126 intra-BLA (basolateral amygdala) treatment without retrieval has no effect on subsequent cue-induced and heroin-induced reinstatement of heroin-seeking behavior. **(A)** Schematic depiction of the experimental procedure. **(B)** Total number of heroin infusions during the acquisition of heroin self-administration. **(C)** Active nosepoke responses during extinction training. **(D)** Active **(left)** and inactive **(right)** nosepokes during the last extinction session and cue-induced reinstatement test. **(E)** Active **(left)** and inactive **(right)** nosepokes during the last extinction session and heroin-induced reinstatement test. *n* = 8 rats per group. Data are means ± SEM. CS, conditioned stimulus; Ext, extinction; Reinst, reinstatement.

In Experiment 3, the same experimental procedure was performed as in Experiment 1, except that U0126 or vehicle infusion was performed without retrieval (see Figure 4A).

#### Experiment 4

The role of delayed ERK inhibition in the BLA after retrieval in subsequent cue-induced and heroin-induced reinstatement of heroin-seeking behavior.

**Figure 5:**
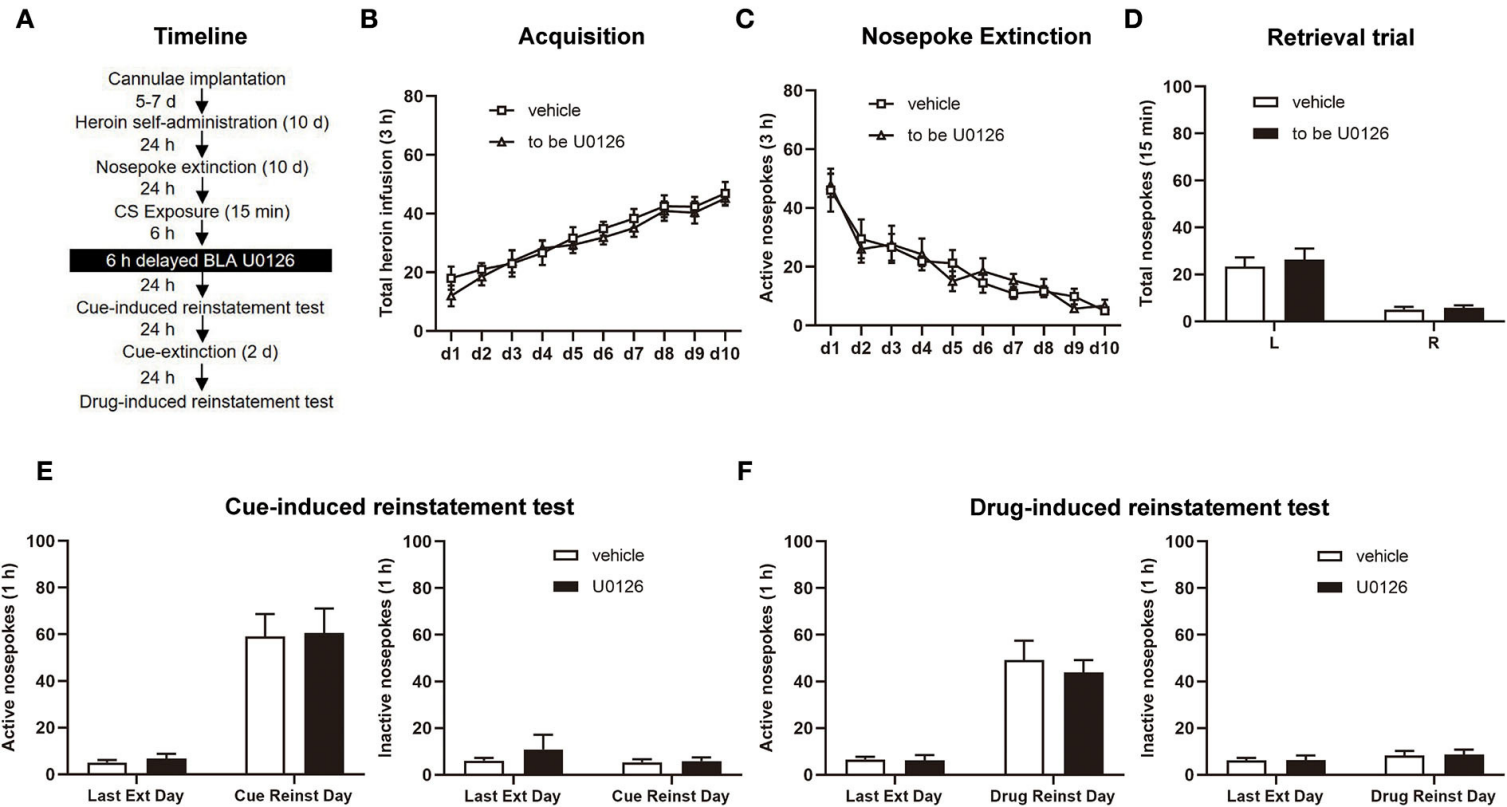
Delayed U0126 intra-BLA (basolateral amygdala) treatment following retrieval has no effect on subsequent cue-induced and heroin-induced reinstatement of heroin-seeking behavior. **(A)** Schematic depiction of the experimental procedure. **(B)** Total number of heroin infusions during the acquisition of heroin self-administration. **(C)** Active nosepoke responses during extinction training. **(D)** Total nosepoke responses during a retrieval trial. **(E)** Active **(left)** and inactive **(right)** nosepokes during the last extinction session and cue-induced reinstatement test. **(F)** Active **(left)** and inactive **(right)** nosepokes during the last extinction session and heroin-induced reinstatement test. *n* = 8 rats per group. Data are means ± SEM. CS, conditioned stimulus; Ext, extinction; Reinst, reinstatement.

We used the same experimental procedure as Experiment 1 in this experiment, except that infusion of U0126 and vehicle was delayed by 6 h after retrieval (see Figure 5A).

### Statistical analysis

Two-way/repeated-measures ANOVAs of GraphPad, v.9.0. were used to analyze the results, which are presented as the mean ± SEM (standard error of mean). Treatment type (U0126 or vehicle) was the between-subjects factor, and test type was the within-subjects factor (last extinction day or cue/heroin-induced reinstatement test; see “**Results**”). Tukey's *post hoc* tests were used to analyze significant differences in specific paired comparisons (*p* < 0.05).

## Results

### Experiment 1: Immediate post-CS ERK inhibition in the BLA reduces subsequent cue-induced and heroin-induced reinstatement of heroin-seeking behavior

To test the role of ERK in the BLA on cue-induced and heroin-induced reinstatement of heroin-seeking behavior, we trained rats in the SA paradigm in experiment 1 (Figure 2A). As the number of total heroin infusions showed, no significant differences were shown between the rats of the vehicle group (*n* = 8) and U0126 group (*n* = 8) in the SA training [main effect of training session: *F*_(9, 126)_ = 20.96, *p* < 0.0001; treatment type: *F*_(1, 14)_ = 0.3410, *p* = 0.5685; training session × treatment type: *F*_(9, 126)_ = 0.1519, *p* = 0.9978; Figure 2B]. In the extinction session, the two groups did not differ from each other in the active nosepoke responses either [main effect of training session: *F*_(9, 126)_ = 23.95, *p* < 0.0001; treatment type: *F*_(1, 14)_ = 1.645, *p* = 0.2205; extinction training × treatment type: *F*_(9, 126)_ = 0.9701, *p* = 0.4680; Figure 2C]. For the retrieval trial, no difference was noticed between the vehicle and U0126 groups in the nosepoke responses [main effect of nosepoke type: *F*_(1, 14)_ = 21.23, *p* = 0.0004; treatment type: *F*_(1, 14)_ = 0.1658, *p* = 0.6900; nosepoke type × treatment type: *F*_(1, 14)_ = 0.1366, *p* = 0.7172; Figure 2D].

A significant difference between the two groups in active nosepokes was revealed by the reinstatement test [main effect of test type: *F*_(1, 14)_ = 54.55, *p* < 0.0001; treatment type: *F*_(1, 14)_ = 15.45, *p* = 0.0015; test type × treatment type: *F*_(1, 14)_ = 18.93, *p* = 0.0007; Figure 2E, left] but not in inactive responses [main effect of test type: *F*_(1, 14)_ = 0.6215, *p* = 0.4437; treatment type: *F*_(1, 14)_ = 0.004233, *p* = 0.9490; test type × treatment type: *F*_(1, 14)_ = 1.398, *p* = 0.2567; Figure 2E, right]. *A post hoc* test was carried out to show a significant reduction in heroin-seeking behavior in the U0126 group compared to the vehicle group (*p* < 0.01; Figure 2E, left). In addition, there was a significant difference between the vehicle and U0126 groups in active responses in the heroin-induced reinstatement test [main effect of test type: *F*_(1, 14)_ = 80.27, *p* < 0.0001; treatment type: *F*_(1, 14)_ = 10.79, *p* = 0.0054; test type × treatment type: *F*_(1, 14)_ = 8.866, *p* = 0.0100; Figure 2F, left] but not inactive nosepoke [main effect of test type: *F*_(1, 14)_ = 0.9777, *p* = 0.3396; treatment type: *F*_(1, 14)_ = 0.07692, *p* = 0.7856; test type × treatment type: *F*_(1, 14)_ = 0.004345, *p* = 0.9484; Figure 2F, right]. A significant reduction in heroin-seeking behavior in the U0126 group compared to the vehicle group was shown by a *post hoc* test (*p* < 0.01; Figure 2F, left column). Therefore, the findings in this experiment suggested that immediate post-CS ERK inhibition in the BLA reduces subsequent cue-induced and heroin-induced reinstatement of heroin-seeking behavior.

### Experiment 2: Immediate post-CS ERK inhibition in the BLA reduces subsequent cue-induced heroin-seeking and the spontaneous recovery of heroin-seeking behavior

In experiment 2, we aimed to investigate whether immediate post-CS ERK inhibition in the BLA has an attenuating effect on the subsequent cue-induced heroin seeking and spontaneous recovery of heroin-seeking behavior after long-term withdrawal (28 days; Figure 3A). Between the vehicle group (*n* = 9) and U0126 group (*n* = 9), no significant difference was observed in heroin SA training [main effect of training session: *F*_(9, 144)_ = 32.17, *p* < 0.0001; treatment type: *F*_(1, 16)_ = 1.036, *p* = 0.3240; training session × treatment type: *F*_(9, 144)_ = 0.7004, *p* = 0.7077; Figure 3B], extinction procedure [main effect of extinction training: *F*_(9, 144)_ = 44.50, *p* < 0.0001; treatment type: *F*_(1, 16)_ = 0.5014, *p* = 0.4891; extinction training × treatment type: *F*_(9, 144)_ = 0.7585, *p* = 0.6548; Figure 3C], or retrieval trial [main effect of nosepoke type: *F*_(1, 16)_ = 25.30, *p* = 0.0001; treatment type: *F*_(1, 16)_ = 0.01829, *p* = 0.8941; nosepoke type × treatment type: *F*_(1, 16)_ = 1.035, *p* > 0.9999; Figure 3D].

In the cue-induced reinstatement test, a significant difference in the two groups was revealed by active nosepoke [main effect of test type: *F*_(1, 16)_ = 55.49, *p* < 0.0001; treatment type: *F*_(1, 16)_ = 8.851, *p* = 0.0089; test type × treatment type: *F*_(1, 16)_ = 12.10, *p* = 0.0031; Figure 3E, left] but not inactive responses [main effect of test type: *F*_(1, 16)_ = 1.884, *p* = 0.1888; treatment type: *F*_(1, 16)_ = 0.2839, *p* = 0.6015; test type × treatment type: *F*_(1, 16)_ = 0.09078, *p* = 0.7671; Figure 3E, right]. The *post hoc* test suggested that there was a significant decrease in active nosepoke in the U0126 group compared to the vehicle group (*p* < 0.01; Figure 3E, left column). In addition, the active nosepoke of the U0126 group in the spontaneous recovery test significantly differed from that of the vehicle group [main effect of test type: *F*_(1, 16)_ = 45.77, *p* < 0.0001; treatment type: *F*_(1, 16)_ = 6.844, *p* = 0.0187; test type × treatment type: *F*_(1, 16)_ = 9.361, *p* = 0.0075; Figure 3F, left] but not inactive nosepoke [main effect of test type: *F*_(1, 16)_ = 0.06534, *p* = 0.8015; treatment type: *F*_(1, 16)_ = 0.04214, *p* = 0.8399; test type × treatment type: *F*_(1, 16)_ = 0.007260, *p* = 0.9332; Figure 3F, right]. A *post hoc* test demonstrated that the active nosepoke of the U0126 group was significantly reduced compared to that of the vehicle group (*p* < 0.01; Figure 3F, left column). The results of experiment 2 suggested that immediate post-CS ERK inhibition in the BLA reduces subsequent cue-induced heroin seeking and spontaneous recovery of heroin-seeking behavior after 28 days of withdrawal.

### Experiment 3: ERK inhibition in the BLA without retrieval has no effect on subsequent cue-induced and heroin-induced reinstatement of heroin-seeking behavior

In experiment 3, we examined the role of ERK in the BLA in cue-induced and heroin-induced reinstatement of heroin-seeking behavior with or without retrieval sessions to investigate whether the attenuating effect of ERK inhibition on reconsolidation of heroin-associated memory is retrieval-dependent (Figure 4A). Consistent with experiments 1 and 2, no significant difference was displayed between the vehicle group (*n* = 8) and U0126 group (*n* = 8) groups in SA training [main effect of training session: *F*_(9, 126)_ = 38.97, *p* < 0.0001; treatment type: *F*_(1, 14)_ = 1.740, *p* = 0.2084; training session × treatment type: *F*_(9, 126)_ = 1.110, *p* = 0.3606; Figure 4B] and extinction [main effect of extinction training: *F*_(9, 126)_ = 17.83, *p* < 0.0001; treatment type: *F*_(1, 14)_ = 0.02481, *p* = 0.8771; extinction training × treatment type: *F*_(9, 126)_ = 1.067, *p* = 0.3915; Figure 4C].

However, different from the results of experiment 1, no significant difference was found in the cue-induced reinstatement test [active nosepoke: main effect of test type: *F*_(1, 14)_ = 63.98, *p* < 0.0001; treatment type: *F*_(1, 14)_ = 0.1758, *p* = 0.6813; test type × treatment type: *F*_(1, 14)_ = 0.1503, *p* = 0.7040; Figure 4D, left; inactive nosepoke: main effect of test type: *F*_(1, 14)_ = 1.072, *p* = 0.3180; treatment type: *F*_(1, 14)_ = 0.1481, *p* = 0.7061; test type × treatment type: *F*_(1, 14)_ = 0.2171, *p* = 0.6484; Figure 4D, right] and the drug-induced reinstatement test [active nosepoke: main effect of test type: *F*_(1, 14)_ = 128.9, *p* < 0.0001; treatment type: *F*_(1, 14)_ = 0.1220, *p* = 0.7320; test type × treatment type: *F*_(1, 14)_ = 0.02509, *p* = 0.8764; Figure 4E, left; inactive nosepoke: main effect of test type: *F*_(1, 14)_ = 0.1982, *p* = 0.6630; treatment type: *F*_(1, 14)_ = 0.1114, *p* = 0.7435; test type × treatment type: *F*_(1, 14)_ = 0.007928, *p* = 0.9303; Figure 4E, right] between two groups. The findings of experiment 3 indicated that the attenuating effect of ERK inhibition in the BLA on reconsolidation of heroin-associated memory is retrieval dependent.

### Experiment 4: Delayed ERK inhibition in the BLA following retrieval has no effect on subsequent cue-induced and heroin-induced reinstatement of heroin-seeking behavior

Finally, experiment 4 tested whether ERK inhibition out the time window of memory reconsolidation attenuates heroin-seeking behavior in the vehicle group (*n* = 8) and U0126 group (*n* =8; Figure 5A). Consistent with experiments 1 and 2, there was no significant difference in the heroin SA training [main effect of training session: *F*_(9, 126)_ = 26.29, *p* < 0.0001; treatment type: *F*_(1, 14)_ = 0.4818, *p* = 0.4989; training session × treatment type: *F*_(9, 126)_ = 0.2792, *p* = 0.9793; Figure 5B], extinction procedure [main effect of extinction training: *F*_(9, 126)_ = 20.64, *p* < 0.0001; treatment type: *F*_(1, 14)_ = 0.009984, *p* = 0.9218; extinction training × treatment type: *F*_(9, 126)_ = 0.4540, *p* = 0.9025; Figure 5C], and retrieval trial [main effect of nosepoke type: *F*_(1, 14)_ = 28.74, *p* = 0.0001; treatment type: *F*_(1, 14)_ = 1.661, *p* = 0.2183; nosepoke type × treatment type: *F*_(1, 14)_ = 0.8740, *p* = 0.3657; Figure 5D].

There was no significant difference between the two groups in cue-induced reinstatement test [active nosepoke: main effect of test type: *F*_(1, 14)_ = 55.11, *p* < 0.0001; treatment type: *F*_(1, 14)_ = 0.04952, *p* = 0.8271; test type × treatment type: *F*_(1, 14)_ = 0.0002967, *p* = 0.9865; Figure 5E, left; inactive nosepoke: main effect of test type: *F*_(1, 14)_ = 0.5720, *p* = 0.4620; treatment type: *F*_(1, 14)_ = 0.8282, *p* = 0.3782; test type × treatment type: *F*_(1, 14)_ = 0.3460, *p* = 0.5658; Figure 5E, right] and the drug-induced reinstatement test [active nosepoke: main effect of test type: *F*_(1, 14)_ = 57.05, *p* < 0.0001; treatment type: *F*_(1, 14)_ = 0.3426, *p* = 0.5677; test type × treatment type: *F*_(1, 14)_ = 0.2215, *p* = 0.6452; Figure 5F, left; inactive nosepoke: main effect of test type: *F*_(1, 14)_ = 1.336, *p* = 0.2671; treatment type: *F*_(1, 14)_ = 0.01948, *p* = 0.8910; test type × treatment type: *F*_(1, 14)_ = 0.004124, *p* = 0.9497; Figure 4F, right]. Thus, the findings of experiment 4 indicated that the attenuating effect of ERK inhibition in the BLA on the reconsolidation of heroin-associated memory is time specific.

## Discussion

Available literature has linked the molecule of the neural mechanism to the behavioral effects of repeated drug administration on learning and memory. Our results demonstrated that inhibition of ERK in the BLA immediately after retrieval interferes with heroin-associated memory in an operant heroin SA paradigm.

Our study first identified that ERK in the BLA is critical for reconsolidation of heroin-associated memory in the classical heroin SA paradigm of relapse. The main findings are as follows: (1) Intra-BLA infusion of U0126 immediately after retrieval significantly attenuated the heroin-seeking behavior induced by cues or heroin in rats. (2) The negative regulation of intra-BLA infusion of U0126 immediately after retrieval on heroin-seeking behavior lasting at least 28 days in rats. (3) Intra-BLA infusion of U0126 but with no retrieval manipulation or infusion with a 6-h delay postretrieval blocked the disruption of the reconsolidation of heroin-associated memory by U0126. In summary, our data showed that intra-BLA infusion of U0126 immediately after retrieval of heroin-associated memory has an inhibitory effect on subsequent heroin-seeking behavior, and the inhibitory effects of U0126 are retrieval-dependent and time-limited.

First, we investigated the role of ERK in the reconsolidation of heroin-associated memory. The results showed that intra-BLA infusion of U0126 during reconsolidation (immediately after retrieval of memory) attenuated cue-induced reinstatement test and lasted at least 28 days relative to vehicle rats (Figures 2, 3), the significance of the current results extending the knowledge of the effect of ERK that interference with the reconsolidation of heroin-associated memory produces a long-lasting effect on heroin-seeking behavior. Consistent with these results, previous studies have revealed the negative effect of ERK inhibition on other forms of memory reconsolidation, including conditioned fear memory (Duvarci et al., 2006), object recognition memory (Kelly et al., 2003; Silingardi et al., 2011), and cocaine memory in both Pavlovian cocaine-related memory and response-outcome associative cocaine-related memory (Miller and Marshall, 2005; Valjent et al., 2006; Wells et al., 2013). Notably, intra-BLA infusion of U0126 exerted a lasting attenuation of the retrieval-dependent heroin-associated memory, which can be interpreted as disrupting reconsolidation that reflects interference by the ERK inhibitor with transcription during the time window when memory is reactivated and is labile (Figure 3). In support of this, in experiment 4, we found that an ERK inhibitor does not affect the consequent cue-induced and heroin-induced reinstatement test 6 h after the retrieval session, the time when reconsolidation presumably occurs (Nader et al., 2000). Our experimental results suggest that the activity of ERK in the BLA regulates the reconsolidation of heroin-associated memory, thus disrupting reconsolidation by the ERK inhibitor U0126 significantly reduces heroin-seeking behavior and prevents relapse.

ERK, a mitogen-activated protein kinase, is activated by various cell growth factors and plays an essential role in cell proliferation and differentiation, leading to meaningful connections to higher learning and memory functions (Adams and Sweatt, 2002; Peng et al., 2010). English and Sweatt (1997) were the first to find the role of ERK signaling in synaptic plasticity that inhibited LTP *via* the ERK inhibitor PD98059 (English and Sweatt, 1997). In support of this in drug addiction, the activity of ERK plays a critical role in the incubation, a phenomenon that enhances cue-induced drug seeking after withdrawal period and demonstrates that ERK phosphorylation increased in the central amygdala (CeA) but not the BLA (Lu et al., 2005; Li et al., 2008). In our study, we did not examine the possible role of the CeA in the effect of ERK inhibition on heroin-seeking behavior since the role of the CeA in the reconsolidation of drug memory has been investigated and was not supported (Wang et al., 2008; Wu et al., 2011). Fortunately, our data suggest that the inhibitory effect of U0126 depends on the BLA, which is consistent with Wells et al. (2013) study that ERK in the BLA is critical for context-response–cocaine reconsolidation (Wells et al., 2013). Consolidation of new drug-related memories is ERK dependent (Lu et al., 2006; Jia et al., 2021). Similar findings have been reported that activation of the ERK pathway results in long-term changes in synaptic activity that underlie the consolidation of new memories (Schafe et al., 2000; Rodrigues et al., 2004; Feld et al., 2005; Lai et al., 2014). Most relevant to our study is that ERK has been reported to be required for memory reconsolidation. In Kelly et al. (2003) demonstrated that ERK in the hippocampal circuitry is required for the reconsolidation of object recognition memory (Kelly et al., 2003). Since then, many laboratories have tested the ability of ERK inhibition to disrupt reconsolidation in other forms of memory, such as auditory fear memory, Pavlovian cocaine-related memory, and instrumental associative cocaine-related memory (Miller and Marshall, 2005; Duvarci et al., 2006; Valjent et al., 2006; Wells et al., 2013). In brief, the above studies hypothesize that ERK may mediate the reconsolidation of heroin-associated memory, regulating various gene expressions to change addictive behavior. Moreover, our data showed that the negative effects of ERK inhibitors on reconsolidation across drug classes are exciting for treating substance use disorders.

Recently, researchers have investigated the role of ERK in reconsolidation-related phenomena. In Rabinovich Orlandi et al. (2020) found that memory reconsolidation is mediated by a “behavioral tagging” process, a behavioral analog of the capture hypothesis and synaptic tagging (Frey and Morris, 1997; Rabinovich Orlandi et al., 2020). Behavioral tagging, whether it acts as a fundamental mechanism underlying drug memory reconsolidation, needs further investigation. Interestingly, a recent study demonstrated that activation of the ERK signaling pathway can change the memory process from reconsolidation to extinction and act as a switch that controls the reconsolidation of fear memory (Fukushima et al., 2021). Furthermore, the ERK 1/2 pathway participates in synthesizing plasticity-related proteins required for spatial memory reconsolidation but is not involved in the tag-setting process. These findings suggest a new strategy that prevents the induction of reconsolidation by activating the ERK signaling pathway.

Together, our data suggest that disruption or modulation of the reconsolidation of heroin-associated memory *via* an ERK inhibitor may serve as a promising option for treating relapse in opiate addicts. This study extends earlier studies showing that ERK activity affects the reconsolidation of drug-related memory, yet its effectiveness in treating clinical populations remains to be tested. It is important to understand further the molecular and cellular mechanisms underlying the reconsolidation of heroin-associated memory to develop target-specific methods for the treatment of opiate addicts.

## Data availability statement

The raw data supporting the conclusions of this article will be made available by the authors, without undue reservation.

## Ethics statement

The animal study was reviewed and approved by the Xiangya Hospital Ethics Committee, Xiangya Hospital (Changsha, China).

## Author contributions

ZC, SC, and HL designed and supervised this study. HL, TH, YZ, ZZ, and QL carried out the main experiments. HL, TH, and YZ prepared the manuscript. ZZ and QL analyzed the data. ZC and SC contributed to manuscript revision with contributions from the other authors. All authors contributed to the article and approved the submitted version.

## Funding

This work was financially supported by National Natural Science Foundation of China (Grant No. 82101247) and the Natural Science Foundation of Hunan Province, China (Grant No. 2021JJ40999).

## Conflict of interest

The authors declare that the research was conducted in the absence of any commercial or financial relationships that could be construed as a potential conflict of interest.

## Publisher's note

All claims expressed in this article are solely those of the authors and do not necessarily represent those of their affiliated organizations, or those of the publisher, the editors and the reviewers. Any product that may be evaluated in this article, or claim that may be made by its manufacturer, is not guaranteed or endorsed by the publisher.
